# What Facial Appearance Reveals Over Time: When Perceived Expressions in Neutral Faces Reveal Stable Emotion Dispositions

**DOI:** 10.3389/fpsyg.2016.00986

**Published:** 2016-06-30

**Authors:** Reginald B. Adams, Carlos O. Garrido, Daniel N. Albohn, Ursula Hess, Robert E. Kleck

**Affiliations:** ^1^Department of Psychology, The Pennsylvania State University, University ParkPA, USA; ^2^Department of Psychology, Humboldt-Universität zu BerlinBerlin, Germany; ^3^Department of Psychological and Brain Sciences, Dartmouth College, HanoverNH, USA

**Keywords:** face perception, emotional expression, person perception, aging, appearance

## Abstract

It might seem a reasonable assumption that when we are not actively using our faces to express ourselves (i.e., when we display nonexpressive, or neutral faces), those around us will not be able to read our emotions. Herein, using a variety of expression-related ratings, we examined whether age-related changes in the face can accurately reveal one’s innermost affective dispositions. In each study, we found that expressive ratings of neutral facial displays predicted self-reported positive/negative dispositional affect, but only for elderly women, and only for positive affect. These findings meaningfully replicate and extend earlier work examining age-related emotion cues in the face of elderly women (Malatesta et al., 1987a). We discuss these findings in light of evidence that women are expected to, and do, smile more than men, and that the quality of their smiles predicts their life satisfaction. Although ratings of old male faces did not significantly predict self-reported affective dispositions, the trend was similar to that found for old female faces. A plausible explanation for this gender difference is that in the process of attenuating emotional expressions over their lifetimes, old men reveal less evidence of their total emotional experiences in their faces than do old women.

## Introduction

“Wrinkles should merely indicate where smiles have been.”

*∼*Mark Twain

Given the importance of emotion recognition for smooth social interaction and interpersonal functioning (cf. Feldman et al., 1991; Carton et al., 1999; Niedenthal and Brauer, 2012) the ability of the elderly to accurately decode emotion expressions has been intensely studied (Ruffman et al., 2008). The question of how accurately the expressions of older individuals are recognized by other human observers, however, and of how emotion perceived in their neutral facial displays may reveal a lifetime of experience and expressed emotion, has received very little empirical attention.

General negative stereotypes may be one source of perceptual bias in reading expressions. Indeed, the most prevalent age-related stereotype is that the elderly are more emotionally negative than their younger counterparts, resulting in an overall negativity bias toward them (Kite and Johnson, 1988; Fabes and Martin, 1991; Ebner, 2008). A meta-analytic review of studies that examined general attitudes about the young and the old (Kite et al., 2005) found that this negative age bias decreases as information about the target person is learned. However, even when this bias is not explicit, it remains substantial when implicit evaluations are examined (see Hummert et al., 2002). Interestingly, such biases also appear to be pan-cultural. For example, in a large study of 26 different cultures, researchers found widespread agreement across cultures regarding negative elderly stereotypes, including physical and socioemotional areas of functionality (Löckenhoff et al., 2009). Further, when Chinese and American cultures were examined, researchers found that contrary to what was expected, both cultures exhibited negative views toward the elderly (Boduroglu et al., 2006). When these same participants were explicitly asked about their emotion expectations (i.e., rating “typical” young and elderly adults, without faces presented), however, these differences were not found.

This perceived negativity extends to the perception of specific expressions in elderly faces. Elderly faces are typically rated as expressing more negative emotions and as being less attractive than young adult faces. Such biases may stem from age-related stereotypes, but another likely source are the wrinkles and folds associated with aging, which can be misperceived as expressing negative emotions (Hess et al., 2012). As such, even an elderly neutral expression may contain incidental expressive features, such as downturned mouth corners, that disrupt and/or bias perception. Such emotion-resembling features then influence perception through a process of emotion overgeneralization (Zebrowitz et al., 2010; see also Todorov et al., 2008 and Adams et al., 2012). These perceptual biases then can serve as both a source of and fuel for general negative elderly stereotypes.

Other social categories such as gender and race have also been found to have facial appearance cues that are confounded perceptually with emotion expressions (Becker et al., 2007; Adams et al., 2015). One particularly compelling study, using a connectionist model trained to detect emotion, revealed that neutral male faces activated angry expression nodes more, and happy expression nodes less, than neutral female faces (Zebrowitz et al., 2010). Likewise, White faces were found to activate anger expression nodes more than African American or Korean faces, while African American faces activated happy and surprise nodes more than White faces. Critically, these findings were based purely on facial metric data, and therefore were necessarily uninfluenced by social learning or culture, thereby offering direct evidence for an objective structural resemblance of typical sex and race appearance to these expressions. Critically, emotion-resembling cues such as these have been demonstrated shape trait impression formation (Adams et al., 2012). Adding emotion-resembling cues (e.g., heightened brow, thinner lips) to otherwise neutral facial texture maps impacted a whole host of trait impressions that are otherwise seemingly independent of emotion (e.g., cooperativeness, honesty, naivety, trustworthiness, dominance, rationality). Despite a growing number of studies now pointing to age-related changes in facial appearance being confounded with expressive cues, little work has been conducted on emotion overgeneralization effects of elderly faces.

Early work conducted by Malatesta et al. (1987b) did suggest that morphological changes in the face due to aging can be misinterpreted as emotional cues due to their direct resemblance to expressions. For instance, drooping of the eyelids or corners of the mouth might be misinterpreted as sadness. In their study, they asked young, middle-aged, and older women to rate the videotaped emotion expressions of young, middle-aged, and older women. One result was that the ability to decode expressions varied with age congruence between encoder and decoder (i.e., the different age groups were better at decoding emotion in same age faces). Most relevant to the current work is that they also found that the emotion expressions of older individuals were more difficult to decode (lower emotion recognition accuracy) due to age-related appearance changes in the face (Malatesta, Izard, Culver, and Nicolich).

More recently, Hess et al. (2012) followed up on this previous work using cutting edge technological advances that offered more precision and experimental control. This work confirmed that advanced aging of the face does degrade the clarity of specific emotional expressions. In this study, identical expressions were applied to young and old faces using FaceGen, a state of the art 3D facial modeling software (Singular Inversions, Vancouver, BC, Canada). The effects of aging were thereby examined while holding the underlying expression constant. In this study, young faces were rated as expressing target emotions more intensely, whereas older faces were rated as more emotionally complex (i.e., they had higher ratings across a number of non-target emotions). In other words, the greater number of emotions present elder faces was associated with a reduced signal clarity for any given target emotion. Neutral old faces were also rated as more emotionally complex, particularly for anger and fear (Hess et al., 2012).

These findings are consistent with another earlier study conducted by Malatesta et al. (1987a), in which they asked 14 elderly models to pose 5 different emotions (anger, fear, sad, joy, and neutral). In this study they found that, aside from happy displays, all other photographic stimuli produced high error rates, suggesting again that wrinkles give rise to more complex and negative looking expressions. Notably, even for neutral faces over 60% of labels given represented negative emotions (note there was no “neutral” label offered): 15% sadness, 14% contempt, 11% anger 8% fear, 7% disgust, 5% guilt, 4% shame/shyness. Matheson (1997) similarly found that when focused on the perception of pain in the face young adult observers were systematically predisposed to see more pain in the faces of the elderly, including in their neutral faces, again presumably due to misreading aging cues as expressive.

One particularly compelling finding in the Malatesta et al. (1987a) study was the correspondence between misperceived emotion displays in elderly faces and the models’ self-reported emotionality. Before posing emotions, the fourteen elderly actors in this study also filled out a Differential Emotion Scale (DES; Izard, 1972) on the same emotions that independent raters later used to label their expressions based on their facial poses (these included, anger, interest, sadness, joy, contempt, disgust, shame/shy, guilt, fear, and surprise). When judges’ mean error rates (i.e., the average error rate for a particular emotion collapsed across all of the actor’s posed expressions) for labeling expressions were examined, they found correlations between specific types of errors and the participants’ own DES scores. For example, judges’ errors for selecting a face as angry predicted participants’ anger scores on the DES, as did sadness, contempt, and guilt. In all, 19 out of 100 correlations conducted were significant, beyond what would be expected by chance alone (i.e., *p* < 0.05). The authors concluded that when individuals make inferences about a face, the errors they make reveal something accurate about the actor’s own emotional predisposition (Malatesta et al., 1987a).

To our knowledge, no study to date has followed up on these intriguing findings. Further these findings only hinted at a possible connection between emotion perceived from neutral faces and the models’ dispositional affect. Thus it remains an empirical question whether the neutral face alone, with all its appearance confounding emotion cues including wrinkles, folds, and facial musculature sagging, can reveal something about the emotional nature of the individual. Thus, we sought to replicate and extend this previous work, and did so in three primary ways.

First, we sought to examine whether these effects generalize to elderly male faces as well. Because there are differences in expected expressivity in males and females, with men being being expected (overall) to suppress emotional expression more than women (see Fabes and Martin, 1991), we might expect old men to show a similar, though reduced effect as has been found for women. Despite an overall expectation to suppress, however, men are also expected to express more power-oriented emotions such as disgust and anger than women (Fabes and Martin, 1991; Fischer, 2000), suggesting that the emotions revealed through a lifetime of expression might also be different for men than women.

Second, Malatesta et al. (1987a) examined misattributed emotion labels to five target displays, to examine whether emotion-resembling cues in the face lead to diagnostically accurate mistakes. Their conclusion was that there is something about neutral facial appearance driving these erroneous, yet accurate impressions. In the current work we wanted to take a more direct approach to this question by focusing on ratings of perceived emotions in neutral faces to assess if these would also predict self-reported ratings. To do this, we used a widely used and well-validated measure of affective disposition, which gauges trait positive and negative affect (i.e., PANAS; Watson et al., 1988). We examined this question using a variety of expression-related face ratings. In Study 1, we had faces rated on two simple scales, one gaging positive and one negative affect. Because these scales were conflated in Study 1, in Study 2 we had the faces rated on the same twenty items that the participants had used to rate themselves – that is on the PANAS items. In Study 3, we extended these findings by having participants rate the faces on a number of discrete indices including “basic” emotions (i.e., the “Big 6” anger, fear sad, joy, disgust, and surprise), as well as a variety of trait dispositions ratings that have been previously linked to emotion resembling cues in the face (Adams et al., 2012). In all three studies the question was the same: does perceived positive/negative expressions in otherwise neutral faces predict the models’ own self-reported positive/negative affect?

The third way in which we sought to extend Malatesta et al.’s (1987a) previous work was to include a young adult sample to serve as a comparison group. If it is the case that age-dependent cues such as wrinkles and folds in the face drive these effects by resembling expressive cues in the face, then we would expect them to emerge most robustly in older faces. Having a young adult control condition then becomes an important baseline comparison to assess this possibility.

In light of research showing that certain age-related cues affect signal clarity by increasing the emotional content perceived in faces, we predicted that the same aging cues that otherwise obscure emotional displays will likewise contribute to perceptions of emotion in a neutral face, and that these emotion perceptions will predict the actual emotional disposition of the models. Below, we begin with a preliminary study that details our procedure for obtaining and validating our stimulus set. We also provide descriptive analyses on the models’ own PANAS scores.

## Preliminary Study: Stimulus Generation

Our current research required that we generate a stimulus set of neutral faces for which we have corresponding self-reported emotion disposition ratings of the models. To do this, we obtained a set of 60 facial images that were captured from videos used in another study (see Huhnel et al., 2014). The photographs depicted White actors who varied in sex and age (30 young and 30 old; 15 of each gender/age group), all of whom also completed the Positive and Negative Affect Schedule (PANAS; Watson et al., 1988).

### Participants

This study was carried out in accordance with the recommendations of the Humboldt-Universität zu Berlin Psychology Department ethics committee. The models in this study were recruited through an internal participant database. All models gave informed written consent and were compensated with 10 Euros for their participation. Models were 30 older (65–94 years; *M* = 72.37 years, *SD* = 6.48; 15 male, 15 female) and 30 younger (20–30 years; mean age = 24.47, *SD* = 3.17; 15 male, 15 female) adults who were screened for neurological or psychiatric disorders. Male and female faces were of equivalent age within each age condition.

### Stimulus Preparation

**FIGURE 1:**
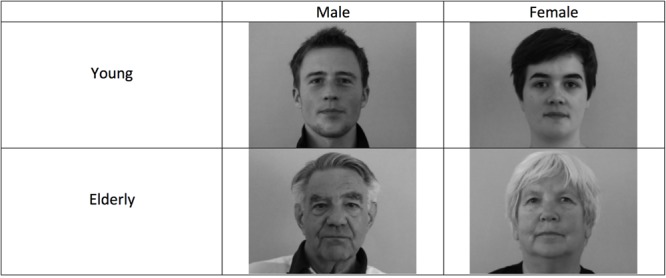
**Sample stimuli from the Humboldt face set used in all studies**.

Photographic frames were captured from dynamic video recordings that featured the models looking directly at the camera as they narrated events in their lives. The models were told to act naturally as they narrated answers to questions that were specifically designed to be as neutral in valence as possible, including questions regarding what they ate for breakfast, to describe their wake up routine, etc. The original videos varied in length, but were all approximately one minute. From these original videos of the actors, a trained assistant then selected a 20 s continuous segment that appeared to be the least expressive. From those shortened segments, co-author Dr. Ursula Hess, who is a gold standard rater (i.e., one of the original coders against whom new coders are tested for certification) in the Facial Action Coding system (FACS: Ekman and Friesen, 1978), selected the photographic frames that best perceptually represented each model’s natural baseline display, selecting frames that also deviated as little as possible from a direct gaze pose. Selected photographs were then cropped and converted to gray-scale (see **Figure 1** for example images).

### FaceReader 6.1^TM^

Because a major premise of the current work is that aging-cues in the face can resemble emotional cues, we also used the 6th version of the software FaceReader (Noldus, 2015) to objectively confirm the neutrality of our stimuli. FaceReader has been well-validated through its use in a growing number of psychological studies showing a high degree of convergent validity with ratings made by human FACS experts (den Uyl and van Kuilenberg, 2005). Its accuracy level in classifying eight emotions (including neutral) is at an average of 89 percent, higher than the rate of emotion recognition by most human subjects (see Lewinski et al., 2014).

FaceReader (version 6.1) models the face using over 500 points, which are based on over 10,000 images that have been manually annotated by experts. Using these points, the face is reconstructed into a virtual mask. An artificial neural network is utilized to estimate which of the six basic emotions (plus neutral and contempt) the face best represents at any given point. The same procedure is used when determining the actor’s age, ethnicity, and sex, which are subsequently taken into account when the algorithm estimates the emotionality present on the face. This work is largely based on Paul Ekman’s FACS (Ekman, 1992a,b,c).

FaceReader is proprietary commercial software. As such, it has a closed access to its code. However, FaceReader is well-developed, having been utilized in over 50 peer-reviewed publications to validate or enhance results, and spanning such diverse fields as psychology, marketing, and methodology. Having been trained on thousands of expressive faces, FaceReader works by detecting a face in an image, identifying 500 landmark points in the face, and then classifying the image according to how likely the emotion is present (or not) in the face (see van Kuilenburg et al., 2005 for a detailed algorithmic description of the FaceReader software). The output consists of coefficients that range from 0 to 1 for each image and for each emotion (including neutrality). Coefficients with higher values indicate a higher likelihood that the given face displays the given emotion (or neutrality).

In our images, young adult images were analyzed using FaceReader’s general module and the elderly adult images were analyzed using FaceReader’s elderly face module, which controls for age-related changes in facial appearance (e.g., wrinkles, folds in skin, and facial musculature sagging). To validate that our stimuli represent baseline neutral poses across all our experimental conditions, we conducted a 2 (age) × 8 (emotion) mixed design ANOVA using the coefficients yielded by FaceReader as the dependent variable and emotion as the within subjects factor. The second factor includes all eight expressive ratings (neutral, happy, sad, angry, surprised, fear, disgust, and contempt). We found a significant main effect of emotion, *F*(7,50) = 184.74, *p* < 0.001, *η*^2^ = 0.99. No effects involving age were significant. So, next we ran a planned comparison of neutral against all other emotions, which revealed that overall the faces were perceived to be more neutral than expressive, *F*(1,59) = 389.9, *p* < 0.001, *η*^2^ = 0.87. Direct comparisons between neutral and each of the seven emotions then revealed that neutrality in these faces, as coded by the FaceReader software, was the predominant display compared to all other possible emotions (all *t*s > 10, all *p*s < 0.001). Means and standard deviations of FaceReader’s output for neutral, by condition is as follows: elderly Males: 0.76 (0.32) Elderly Females: 0.80 (0.29) Young Males: 0.83 (0.26) Young Females: 0.86 (0.20). As indicative from the above coefficients, FaceReader scored all the faces, regardless of age group and sex, as appearing highly neutral. Further, FaceReader is able to predict actual age of faces with a high rate of accuracy. We used this to examine whether the faces here varied in age-related appearance across our gender conditions. FaceReader’s predicted age and the models’ actual age were highly correlated (*r*(58) = 0.76, *p* < 0.001), and critically neither varied across our gender conditions. From this we can conclude that our young and old models were matched across for actual and perceived age across gender conditions.

### PANAS Scores

All models filled out the 20-item PANAS twice, once before the filming took place, and once after. The 10 positive and 10 negative PANAS traits were then combined to create standardized measures of positive (PA) and negative (NA) affective states for each of the two time points. PANAS scores obtained at both time points correlated highly with one another, PA (*r* = 0.57, *p* < 0.001) and NA (*r* = 0.78, *p* < 0.001). Because this scale is a highly reliable trait measure (see Crawford and Henry, 2004 for extensive evaluation of this widely used instrument), we combined scores to best approximate each individual’s central tendency in rated emotional dispositions. We then ran a 2 (age: young/old) × 2 (gender: male/female) × 2 (affective type: PA/NA) repeated measures ANOVA to examine any possible differences between the stimulus groups on PA/NA scores. The only effect to reach significance was a main effect of affect type, such that participants across all groups reported more positive (*M* = 29.37, *SD* = 2.98) than negative affect (*M* = 12.9, *SD* = 2.01), *F*(1,14) = 254.74, *p* < 0.001, *η^2^* = 0.95. Thus, the individuals in our four conditions (old men, old women, young men, and young women) did not vary in their self-reported dispositional affect. The final set of 60 photographs and PANAS scores were then used in the three studies reported below, and because variation in expressive resemblance of facial appearance was the primary focus, all analyses reported in these studies are at the items level.

## Study 1

The purpose of Study 1 was to examine whether independent ratings of our non-expressive models’ faces on two scales, positive and negative affect, are positively associated with the models’ own self-reported positive affect (PA) and negative affect (NA) as measured by the PANAS. Participants were specifically instructed to attend to expressive cues in the face. They were asked to rate how much each face displayed was currently *expressing* positive and negative affect. This was to ensure that our human raters were tuned to the emotion resembling aspects of facial appearance. Then we examined the association of these ratings with the models’ self-reported emotion dispositions.

### Methods

#### Participants

Studies 1–3 were carried with the approval of Penn State’s Institutional Review Board for Human Subject Research. All participants gave informed written consent before participating and were compensated with partial course credit for their participation. Undergraduate students enrolled in psychology classes were recruited via the departmental participant pool’s online recruitment system. In all studies we recruited undergraduate students (typically ranging from 18 to 24 years of age) who were enrolled in psychology classes. Participants were recruited via the departmental participant pool’s online recruitment system. For Study 1, forty participants (12 men) participated in the study in exchange for class credit. Twenty-seven participants identified as White, 6 as Black, 2 as Latino, 4 as Asian, and 1 multiracial.

#### Design and Procedure

The purpose of the study was described as an examination of perceptions of people’s mental states based on their faces. After completing the informed consent process, participants were instructed to read the instructions carefully before beginning the survey. Instructions informed participants that they were to rate 60 faces one by one and urged them to go with their first instinct and to not deliberate excessively over any of the faces or of the expressive states they rated. In all three studies, images were displayed subtending a visual angle of approximately 7.6° × 5.1. As is the case for all studies reported on this paper, participants completed the task in groups of up to six in order to maximize efficiency and reduce data collection time. However, each participant was assigned to a computer workstation and workstations were separated by partitions. First, participants were asked to rate the extent to which each of the 60 faces *expressed* positive and negative affect (on separate scales) using a scale ranging from “0” to “100,” where 0 represented lowest degree of the type of affect and 100 represented the highest degree. Positive affect was defined as “a mood dimension that consists of specific pleasant or positive emotions.” Negative affect, on the other hand, was defined as “the full spectrum of negative or unpleasant emotions.” Each participant rated all 60 photographs and each photograph remained on the screen until both positive and negative affect ratings were made. The order of presentation of the stimuli was randomized and the presentation of the affect scales (positive versus negative affect) was counterbalanced across participants. Lastly, participants provided demographic information and were fully debriefed.

### Results

#### Affective Perception of Faces

We first assessed the correlation between rated perceptions of positive and negative affect. Because the two scales correlated very highly (*r* = 0.96, *p* < 0.001), we reverse scored the negative affect scale to create a composite score that ranged from very negative (low numbers) to very positive (high numbers). We then conducted a 2(gender: male/female) by 2(age: young/old) factorial within-subjects ANOVA to examine differences of affective attributions among the groups. As predicted, there was a main effect of age, *F*(1,56) = 6.93, *p* = 0.01, *η*^2^ = 0.11, such that elderly targets (*M* = 43.28, *SD* = 16.93) were rated as more negative/less positive than young targets (*M* = 54.57, *SD* = 15.89), *t*(57) = 2.63, *p* = 0.01. No other effects reached significance.

#### Relation to Self-Reported PANAS Scores

**FIGURE 2:**
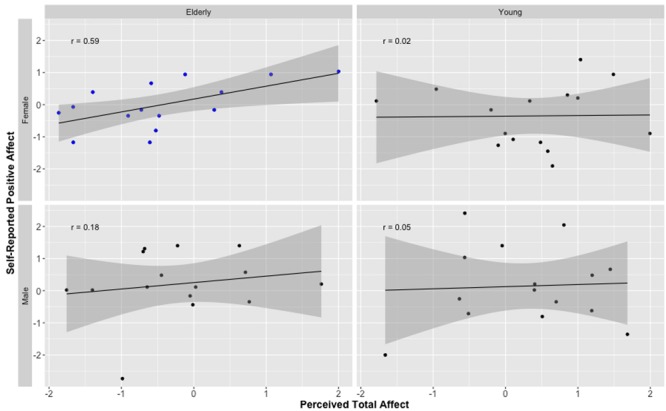
***Z*-score-transformed correlation coefficients demonstrating the relationship between perceptions of positive affect and self-reported positive affect (from PANAS) for elderly and young male and female faces.** *r* is Pearson correlation. Shaded area represents standard error. Independent ratings of positive affect are the combined ratings of positive affect and the reversed scored ratings of negative affect. Only the correlation for elderly female faces is significant, all other coefficients *p* > 0.05.

Next, we computed correlations between the independent ratings of target facial expression and the targets’ own self-reported affect using the composite PANAS scores. As predicted, independent ratings of perceived affect when viewing elderly target faces was positively associated with the targets’ self-reported positive affect (*r* = 0.36, *p* = 0.05). This was not, however, the case for self-reported negative affect (*r* = –0.01, *p* = 0.97). Affective ratings of young adult faces predicted neither self-reported NA (*r* = 0.04, *p* = 0.84), nor PA (*r* = 0.03, *p* = 0.89). Finally, when analyzed separately for each gender, we found that the significant correlation between affective ratings of the elderly target faces and target self-reported PA scores was primarily carried by perceptions of elderly female faces (*r* = 0.59, *p* = 0.01); see **Figure 2**. Elderly male faces showed a positive association, but this did not reach significance (*r* = 0.18, *p* = 0.53).

## Study 2

Because positive/negative ratings scales were statistically conflated in Study 1, in Study 2 we had the same faces rated on the entire PANAS battery. The PANAS is a collection of 20 emotion items, half loading on positive and half on negative affect factors. This instrument was specifically designed, through the use of multiple item ratings, to differentiate and thus statistically separate positive/negative affect ratings. Thus, in this study we aimed to replicate and extend the findings in Study 1 by examining positive and negative affect ratings of the faces as distinct dimensions of perceived emotionality.

### Methods

#### Participants

One hundred thirty one undergraduate-aged participants (36 men) participated in exchange for partial class credit. Five participants identified as White, 8 as Black, 5 as Latino, 18 as Asian, and 5 as multiracial.

#### Design and Procedure

Participants rated the extent to which the 60 faces *expressed* each the 20 emotion items included in the PANAS. Of the 20 affective states, 10 were positive in valence (e.g., interested, enthusiastic) and 10 were negative (e.g., distressed, scared). Due to the large number of state ratings, we collected data in three waves. Waves differed only in the stimuli presented to participants and each wave contained 20 of the 60 total stimuli. For each wave, stimuli selection was based on random assignment without stimulus replacement. Each wave was conducted subsequent to the end of the previous wave and the data collection period lasted less than a month. As in the previous study, the order of both presentation of the stimuli and of the emotional expression scales, was randomized but expression scales were presented on the same survey screen. Otherwise, instructions, procedures, and stimuli were identical to those used in Study 1.

### Results

#### Affective Perception of Faces

As prescribed for the PANAS, we averaged the scores of the 10 positive and 10 negative items to generate positive and negative affect scores. Both the positive (α = 0.95) and negative (α = 0.92) affect composites achieved high reliability. There was a high correlation between the two variables (*r* = –0.81, *p* < 0.001). Thus, we again reverse scored the negative affect ratings and combined them with the positive affect scores to create a single affect index. As before, these scores ranged from negative to positive (high scores indicate greater positivity). First, we conducted a 2(gender) by 2(age) factorial ANOVA on these scores. A marginally significant main effect of age emerged, *F*(1,56) = 2.95, *p* = 0.09. Consistent with the results of Study 1, the elderly faces (*M* = 51.12, *SD* = 7.70) received lower ratings of positive/higher negative affect than the younger faces (*M* = 54.69, *SD* = 8.30).

#### Relation to Self-Reported PANAS Scores

**FIGURE 3:**
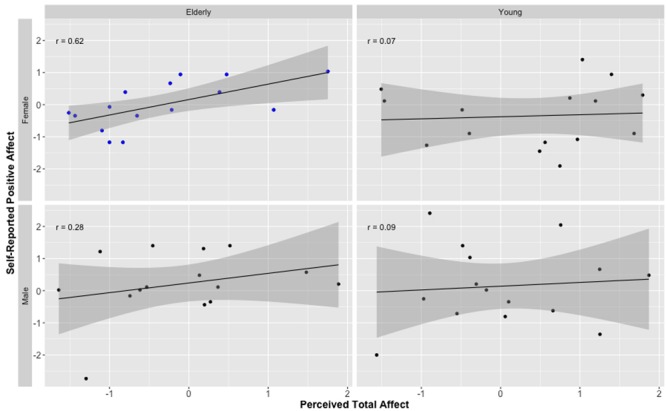
***Z*-score-transformed correlation coefficients demonstrating the relationship between perceptions of positive affect from neutral faces and self-reported positive affect (from PANAS) for elderly and young male and female models.** *r* is Pearson correlation. Shaded area represents standard error. Independent ratings of positive affect are the combined ratings of positive affect and the reversed scored ratings of negative affect. Only the correlation for elderly female faces is significant, all other coefficients *p* > 0.05.

First, we conducted correlations between attributions and self-reported affect by age group. Replicating the results of Study 1, ratings of positive affect for elderly targets were positively associated with self-reported PA (*r* = 0.42, *p* < 0.05). This association was not apparent for face ratings and self-reports of young targets (*r* = 0.04, *p* = 0.85). As in Study 1, when analyzing the data separately for each gender, we found that the significant association between perceptual ratings and self-reports of positive affect was primarily driven by elderly females (*r* = 0.62, *p* = 0.01); see **Figure 3**. Again, this association was positive for elderly male targets as well, but did not reach significance (*r* = 0.15, *p* = 0.59). No other correlations reached significance for any of the other target groups or affect type (positive or negative).

## Study 3

We aimed to replicate and extend this work to basic emotions (e.g., anger, fear, sad, and happy), as well as trait impression ratings known to be derived from emotion resembling features of the face (e.g., trustworthy, dominance; see Said et al., 2009; Adams et al., 2015). Because principal components analysis (PCA) revealed that both sets of items yielded a primary valence factor, we included composite results for each for comparison and conceptual continuity with the prior two studies. However, because these ratings are also widely used as independent predictors, we also report each item’s association with the stimulus models’ PANAS scores separately.

### Methods

#### Participants

Forty-two undergraduate-aged participants (15 men) enrolled in psychology classes participated in the study in exchange for class credit. Thirty participants identified as White, 2 as Black, 2 as Latino, 5 as Asian and 3 as multiracial.

#### Design and Procedure

Instructions, procedures, and stimuli used were identical to those used in the previous studies with the exception of the type of ratings participants performed. For this study, participants rated the extent to which each face appeared to express six primary emotions (happy, sad, joy, surprise, disgust, anger) and to possess five traits (affiliative, attractive, dominant, threatening, trustworthy). As in the previous study, presentation of the stimuli and ratings were randomized. As with all studies reported on this paper, participants made their ratings of the five emotions and five traits using a 0 “lowest degree” to 100 “highest degree” scale.

### Results

#### Affective Perception of Faces

We first examined correlations of all six primary emotions. With the exception of surprise, each emotion was highly correlated with all other emotions (all *r*s > ± 0.34, all *p*s < 0.01; see **Table 1A**). Given the high degree of intercorrelations between these emotions, with each associated with a clear positive or negative valence, we performed a PCA to determine whether valence was an explanatory factor. The scree-plot revealed a one-factor solution (i.e., only one factor emerged with eigenvalue greater than 1), which explained 71% of total variance. As expected, the component matrix yielded positive loadings for anger, disgust, fear, sadness, and a negative loading for joy on the principal component (see **Table 2A**). Thus, we converted all the emotion ratings to *z*-scores before reverse scoring the negative emotions and converting all five into one composite emotion score that, like in Studies 1 and 2, ranged in valence from negative to positive. We then ran a 2(gender) × 2(age) between subjects ANOVA on the distribution of *z*-scores. As expected, there was a main effect of age, *F*(1,56) = 9.82, *p* < 0.01, *η*^2^ = 0.15, such that elderly targets (*Z* = –0.32) were rated as appearing less positive in their affect than were young targets (*Z* = 0.32), *t*(57) = 3.11, *p* < 0.01.

**Table 1:** Intercorrelations between Emotion (A) and Trait (B) ratings of stimulus items (Study 3).

(A) Emotions	1	2	3	4	5	6
(1) Anger	–	0.34^∗∗^	0.47^∗∗^	-0.76^∗∗∗^	0.89^∗∗∗^	-0.22
(2) Fear		–	0.66^∗∗∗^	-0.65^∗∗∗^	0.42^∗∗∗^	-0.26^∗^
(3) Sadness			–	-0.75^∗∗∗^	0.51^∗∗∗^	-0.24
(4) Joy				–	-0.82^∗∗∗^	-0.05
(5) Disgust					–	-0.11
(6) Surprise						–

**(B) Traits**	**1**	**2**	**3**	**4**	**5**	

(1) Affiliative	–	0.63^∗∗∗^	-0.12	-0.39^∗∗^	0.66^∗∗∗^	
(2) Attractive		–	-0.39^∗∗^	-0.60^∗∗∗^	0.65^∗∗∗^	
(3) Dominant			–	-0.83^∗∗∗^	-0.52^∗∗∗^	
(4) Threatening				–	-0.83^∗∗∗^	
(5) Trustworthy					–	

*^∗^*p* < 0.05, ^∗∗^*p* < 0.01, ^∗∗∗^*p* < 0.001.*

**Table 2:** Principal components factor solutions for basic emotion (A) and trait impression (B) ratings (Study 3).

(A) Emotions	Factor 1 (Valence)	
Eigenvalue	3.537	
Anger	**0.84**	
Disgust	**0.88**	
Fear	**0.71**	
Sadness	**0.80**	
Joy	**–0.95**	

**(B) Trait impressions**	**Factor 1 (Valence)**	**Factor 2 (Power)**

Eigenvalue	3.296	1.056
Affiliative	**0.94**	-0.01
Attractive	**0.79**	-0.36
Dominance	-0.06	**0.96**
Threatening	-0.41	**0.89**
Trustworthiness	**0.72**	-0.57

*Because only one factor emerged for basic emotions there was no Varimax rotation. Trait extraction method involved Varimax with Kaiser Normalization. Variables loading on each factor are indicated in bold.*

#### Relation to Self-Reported PANAS Scores

**FIGURE 4:**
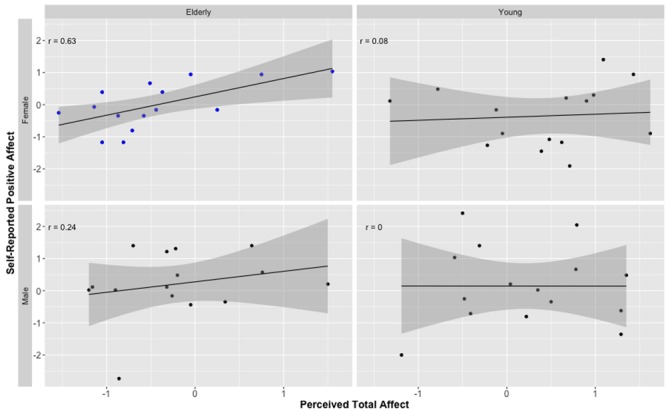
***Z*-score-transformed correlation coefficients demonstrating the relationship between perceptions of positive affect and self-reported positive affect (from PANAS) for elderly and young male and female faces.** *r* is Pearson correlation. Shaded area represents standard error. Independent ratings of positive affect are the combined attributions of joy and reversed scored ratings of anger, fear, disgust, and sadness. Only the correlation for elderly female faces is significant, all other coefficients *p* > 0.05.

Next, we examined whether ratings of positive/negative emotions to the targets correlated with self-reported affect on the PANAS for each of the two age groups. As in Studies 1 and 2, the correlation between the emotion valence index and self-reported positive affect was significant for elderly targets (*r* = 0.37, *p* < 0.05), but not for young targets (*r* = –0.03, *p* = 0.87). Again, there was no association between the emotional valence index of faces and self-reported negative affect either for the elderly (*r* = 0.00, *p* = 0.99) or the young (*r* = 0.03, *p* = 0.87). Lastly, splitting the analyses by gender of target revealed once again that the significant association between positive/negative emotion perceived from elderly faces and self-reported PA was driven primarily by elderly female targets (*r* = 0.58, *p* < 0.05); see **Figure 4** (see also **Table 3A** for independent correlations between each emotion and self-reported PANAS scores). Elderly male targets again showed a positive association, which did not reach significance (*r* = 0.20, *p* = 0.47).

**Table 3:** Correlations between emotion and trait ratings that loaded on the valence factor of the principal components analysis (PCA) and elderly female models’ self-reported PA (Study 3).

(A) Emotion ratings	
Anger	-0.29
Disgust	-0.36
Fear	-0.67**
Sadness	-0.58*
Joy	0.58*

**(B) Trait ratings**	

Affiliative	0.57^†^
Attractive	0.67**
Trustworthiness	0.46

^†^p < 0.1, ^*^p < 0.05, ^**^p < 0.01.

#### Trait Perception of Faces

Because the emotions perceived in neutral faces have been directly implicated as influencing impression formation (e.g., Adams et al., 2012), we also performed a PCA on the five trait ratings using varimax rotation with Kaiser normalization (see **Table 1B** for intercorrelations of traits). The scree-plot revealed a two-factor solution (both factors with eigenvalues above 1). The first factor included items highly related conceptually to the construct of valence, and the second had items corresponding to a power/dominance dimension, another common factor found in the emotion and person perception literature (Todorov et al., 2008). The first factor explained 62% of total variance that formed a “positivity” dimension. The second factor explained 21% of the variance, included the other two traits (dominance and threatening) to form a “power” dimension (see **Table 2B**). Consequently, we *z*-scored and averaged corresponding traits to create an index of “positivity” and an index of “power.”

A 2(gender) by 2(age) factorial ANOVA using the positivity index as the dependent variable yielded a significant main effect of age, *F*(1,56) = 8.26, *p* < 0.01, *η^2^* = 0.13, such that the elderly were seen as less positive (*M* = 24.25, *SD* = 5.55) than the young (*M* = 29.5, *SD* = 8.86). There was also a marginal main effect of gender, *F*(1,56) = 3.14, *p* = 0.08, *η*^2^ = 0.05, such that female faces were rated as more positive (*M* = 16.42, *SD* = 8.68) than male faces (*M* = 15.25, *SD* = 6.55). These main effects were qualified by a significant interaction, *F*(1,56) = 4.35, *p* < 0.05, *η*^2^ = 0.07. Simple effects comparisons showed that young women (*M* = 33.02, *SD* = 8.66) were rated greater in positivity than both elderly women (*M* = 23.97, *SD* = 6.09), *t*(57) = 3.51, *p* = 0.001 and young men (*M* = 25.98, *SD* = 7.81), *t*(57) = 2.73, *p* < 0.01.

Using the power index as the dependent variable in the factorial ANOVA yielded a main effect of sex, *F*(1,56) = 8.58, *p* < 0.01, *η*^2^ = 0.13, and of age, *F*(1,56) = 7.13, *p* = 0.01, *η*^2^ = 0.11, but no interaction, *F*(1,56) = 0.64, *p* = 0.43. Elderly targets (*M* = 30.98, *SD* = 9.56) were rated as more powerful than young targets (*M* = 24.78, *SD* = 9.49), *t*(57) = 2.67, *p* = 0.01, and men (*M* = 31.28, *SD* = 9.25) were rated as more powerful than women (*M* = 24.48, *SD* = 9.59), *t*(57) = 2.93, *p* < 0.01.

#### Relation of Trait Ratings to Self-Reported PANAS Scores

Overall the correlation between the index of trait positivity and self-reported positive affect was not significant for elderly, *r* = 0.18, *p* = 0.33, or young, *r* = –0.10, *p* = 0.61, targets. However, conducting the analyses separately for each gender revealed a positive correlation between the trait positivity index and self-reported positive affect for elderly women, *r* = 0.60, *p* < 0.05 (see **Table 3B** for independent correlations between each related trait and self-reported PANAS scores). No significant correlations were observed between the power index and self-reported PANAS scores for any of the target groups (all *r*s < ± 0.15).

## General Discussion

Even though self-reported PANAS scores showed no age or gender differences in actual positive and negative affective styles for the models (Preliminary Study), across all three subsequent experimental studies we found a strong perception of more negative affect expressed by elderly faces compared to young faces. This bias was apparent for single item positive/negative ratings (Study 1), the full set of PANAS face ratings (Study 2), and emotion profile and trait impressions indices (Study 3). That elderly faces here were seen as expressing more negativity fits with what has been reported in the previous research (Malatesta et al., 1987a; Matheson, 1997; Hess et al., 2012) suggesting that age-related cues in the face (drooping around the eyes, wrinkles, folds) are at least partially confounded with emotion expressions, which in turn influence perception.

Importantly, the primary question addressed in the current research was whether these age-related appearance cues also convey accurate information about the target’s actual emotional disposition. Studies 1–3 consistently revealed that perceptions of elderly faces did accurately predict the models’ own self-reported affective dispositional styles, but only on the positive affect dimension of the PANAS, not on negative affect. In addition in all three studies it was ratings of elderly female faces that drove the effects. Critically, this effect was not apparent in ratings of young adult faces.

The previous related work (Malatesta et al., 1987a) only included perceptions of elderly female faces. Thus, the current work replicates this previous research while at the same time delimiting its generality to female stimuli. To our knowledge this is the first replication of this work since it was originally reported. The current results extend these previous findings in a number of important ways. First, we included young adult faces, which do not have age-related changes such as wrinkles. Across all studies, we found no evidence that these faces convey information that is diagnostic of self-reported emotional disposition, lending additional credence to the conclusion that the effects we found are due to age-related changes in the face. Because we had raters in each study focus specifically on expressive aspects of the faces to make their ratings, these findings are consistent with previous suggestions that there are age/emotion cue confounds. We further extended this work by including a sample of elderly men. Across all three studies, ratings of old male faces did not significantly predict self-reported emotionality, though the trend was in the same direction as that found for old female faces.

Helping explain these differences, a prevailing gender stereotype across cultures is that women are more “emotional” than men in that they are expected to feel and express emotions more than men (Brody and Hall, 2000; Shields, 2000). Directly relevant to the current work is evidence that gender-based expectations are particularly pronounced for emotional expression (Fabes and Martin, 1991), and that these stereotypes drive gender differences in emotional expression. Based on this, Fabes and Martin posited a deficit model of male expressivity, which essentially underscores the tendency for males to be stoic even in the face of intensely felt emotion. They suggest that while males may experience a similar amount of emotion as females, they are expected to suppress or inhibit their expression of it. They conclude (1991, p. 539): “With few exceptions, it appears that the stereotype that females are more emotional than males is based on the deficit model of male expressiveness (i.e., a belief that males do not express the emotions they feel).” In the same vein Shields (2005) describes emotional restraint as the culturally valued expressive mode for men, suggesting that even if emotions are shown, this should ideally be in a restrained form (Shields, 2005). The consequent reduced expressivity helps explain the lack of significant effects emerging for elderly men. If men are less likely to express emotion, expression is less likely to influence their aging cues in the face.

Whereas men are expected (and tend) to be less expressive, with a neutral mask being their default expression (Fabes and Martin, 1991; Fischer, 1993), women are expected to be emotionally expressive, particularly with regard to happiness, fear, and sadness (Fabes and Martin, 1991; Briton and Hall, 1995). There exists particularly strong evidence that women smile more often than men. Indeed, a study examining yearbook pictures shows that on average 80% of the women but only 55% of the men smiled (Dodd et al., 1999). These findings suggest that women may feel obliged to smile. A failure to smile socially may be met with disapproval, because it defies the affiliative role women are expected to adhere to (LaFrance et al., 2003). In fact, women expect more costs when not expressing positive emotions in an “other” oriented context (Stoppard and Gruchy, 1993) and are rated more negatively when they do not smile (Deutsch et al., 1987).

This same idea was well typified in the landmark paper “Perfidious Feminine Faces,” by Bugental et al. (1971), who reported that children perceived verbal messages from their fathers, when delivered with a smile, as more positive than when delivered without a smile. Verbal messages from their mothers, however, were perceived as no more positive when coupled with a smile than when not. This finding is consistent with the conclusion that smiling is the default expression for women. Thus, for women to convey genuine positivity presumably would require far more intense and frequent smiling behavior. Perhaps, then, it should be no surprise that even young adult women have higher smile lines when smiling, and thicker zygomaticus major muscles than men (measured using ultrasound: McAlister et al., 1998).

Critically, this smiling behavior in women has been found to predict real-life outcomes as well. In one study examining photographs of women in yearbooks, it was found that smiles by women that appeared more genuine (i.e., Duchenne smiles) predicted life satisfaction scores up to 25 years after the picture was taken (Harker and Keltner, 2001). Specifically, women whose smiles appeared more genuine were more achievement focused, organized, more approachable, and less susceptible to negative emotions than those who showed less. Overall we found that aging cues in elderly faces are misperceived as expressing more negative emotion compared to young faces. That we then found that ratings of elderly women’s faces predicted positive affect is interesting, but not contradictory. Notably, both ratings of negative and positive expressivity in elderly women’s faces predicted their self-reported positive affect. Thus variability across faces is due to both. Perceived negative emotion in elderly neutral faces predicted low self-reported positivity, and vice versa. Such age-related appearance across elderly female models’ faces was highly predictive of accurate self-reported positive disposition across all three studies.

The tendency for women to smile more often, and presumably to be obliged to smile even more intensely to convey genuine positivity, may help explain why it was only dispositional positive affect that was predicted from their faces in the current studies, not negative affect, as smile related wrinkles may temper the negative-resembling expressions caused by aging in the face. If men are prone to suppress outward expressions, then their expressiveness would not have as noticeable impact on facial appearance over time. Likewise, if women smile so much more often than men, this too would presumably reveal itself in the face over time. Notably, elderly women were not rated as looking any more positive than elderly men (or young men and women) overall, but variation in positivity that was perceived in their faces did reveal actual self-rated dispositional positivity.

Although there are gender differences in overall expectations for emotional expression, these expectations also appear to be divided along the dimensions of dominance and affiliation (Adams et al., 2015). Women are expected to express more overall emotion in comparison to men with some notable exceptions. That is, men are expected to inhibit “weak” emotional expressions, but to express powerful emotions, such as anger and disgust (Fabes and Martin, 1991; Fischer, 2000). The current set of studies did not focus primarily on power-oriented emotions, and so perhaps missed meaningful facial cues that would have been more predictive for men. In this vein, we conducted a *post hoc* analysis of face ratings of anger and disgust in Study 3, which did predict self-reported hostility on the PANAS (*p*s < 0.05). These two effects, however, do not survive corrections for multiple tests, but they are suggestive that future work focused more on male-oriented “power” emotions might be a fruitful avenue for continued work in this domain when examining aging in the male face.

With recent evidence that perceiving emotion-resembling cues in otherwise neutral facial appearance serves as a powerful mechanism of impression formation, these results highlight new vistas of exploration in person perception. Malatesta et al. (1987a; p. 68) suggested that “the “misattributions” of decoders are also probably in part a consequence of the leakage of prepotent emotion response tendencies.” It is also possible that the face carries with it emotional residue from one expressive experience to another that reveals current, previous, or chronic emotional states. Such insights could eventually help explain how it is that we can extract accurate perceptions of others just by viewing their faces. It has been shown, for instance, that people can accurately identify political-race winners (Todorov, 2005; Rule et al., 2010a), political affiliation (Rule and Ambady, 2010), business leaders’ salaries (Rule and Ambady, 2011), sexual orientation (Rule et al., 2008), and religious affiliation (Rule et al., 2010b). Recently, Tskhay and Rule (2015) specifically implicate emotional processes underlying such effects, demonstrating that emotions are embedded in the very mental representations people have of certain social groups. Thus, if people are primed to signal or detect their social group members, emotional leakage or residue in the face may be means through which that information is gleaned, even if unintentionally.

In sum, there may be partial truth to the age-old warning “if you don’t stop making that expression, your face will freeze that way!” Our findings suggest that at least over the course of a lifetime, expression can become etched into the folds and wrinkles of the face and become a stable part of a person’s neutral visage, offering diagnostic information of their dispositions to others. While it is certainly possible that extraneous idiosyncratic features (genetics, tanning, or plastic surgery) may also influence the perception of a neutral face, we believe that expressions—a behavior present since birth—hold equal, if not more powerful influence over the evolution of the face as it ages. Cicero said it well: “The face is a picture of the mind with the eyes as its interpreter.” Our findings suggest that even when presumably neutral, the face may well reveal more to the eyes than previously realized.

## Author Contributions

All authors listed, have made substantial, direct and intellectual contribution to the work, and approved it for publication.

## Conflict of Interest Statement

The authors declare that the research was conducted in the absence of any commercial or financial relationships that could be construed as a potential conflict of interest.

The reviewer LK declared a shared affiliation, though no other collaboration, with one of the authors UH to the handling Editor, who ensured that the process nevertheless met the standards of a fair and objective review.
